# The health benefits of exercise therapy for patients with Down syndrome: A systematic review

**DOI:** 10.4102/ajod.v8i0.576

**Published:** 2019-10-23

**Authors:** Yvonne Paul, Terry J. Ellapen, Marco Barnard, Henriëtte V. Hammill, Mariëtte Swanepoel

**Affiliations:** 1 Department of Sport and Dental Therapy, Faculty of Health Science, Tshwane University of Technology, Cape Town, South Africa; 2 School of Biokinetics, Recreation and Sport, Physical Activity Sport and Recreation (PhASRec), North-West University, Potchefstroom, South Africa

**Keywords:** cardiometabolic, Down syndrome, exercise, muscle strength, proprioception, obesity

## Abstract

**Background:**

Many patients with Down syndrome (PWDS) have poor cardiometabolic risk profiles, aerobic capacities and weak hypotonic muscles, primarily because of physical inactivity and poor diet.

**Objectives:**

This study discusses the benefits of exercise therapy on body composition, aerobic capacity, muscle strength, proprioception and cardiometabolic profiles of PWDS.

**Methods:**

A literature review using the Crossref metadatabase, following Preferred Reporting Items for Systematic Reviews and Meta-Analyses (PRISMA), focusing on the period 2007-2018, was undertaken. Each record was judged adopting the modified Downs and Black Appraisal Scale. The literature investigation identified 15 701 records. Records were excluded if they were published before 2007, pertained to the impact of exercise on intellectual disabilities beyond Down syndrome or the impact of medical, pharmaceutical, nutrition and psychological interventions among PWDS and were published in languages besides English. Nineteen articles were synthesised into this commentary.

**Results:**

PWDS have a heightened cardiometabolic risk profile and high oxidative stress associated with elevated insulin resistance, poor insulin sensitivity, atherosclerosis and hypertension. PWDS have low aerobic capacity (VO_2max_), peak heart rates, muscle strength, agility and balance. Regular physical activity is beneficial to improve their VO_2max_ and muscle strength. Moreover, regular physical activity reduces lipid peroxidation and arterial cell wall damage, the pathogenesis of atheroma is limited.

**Conclusion:**

Exercise therapy compliance seems to have a positive impact on the cardiometabolic risk profile, muscle strength and aerobic work capacity of PWDS. Nonetheless, additional vigorous experimental investigations are necessary to better understand the effect of exercise therapy on the aerobic, strength, proprioception and cardiometabolic risk profile of PWDS.

## Introduction

The longevity of patients with Down syndrome (PWDS) has chronologically extended over the course of the last century (Vis et al. 2009). In 1929, the average lifespan of PWDS was 9 years; this later increased to 12 years (1949), and then progressively extended to 35 years (1982) and reaching 55 years (2007) a decade ago (Barnhart & Connolly 2007). The increased longevity among PWDS is quite possibly because of improved medical and pharmaceutical management (Duffels et al. 2009). Vis et al. (2009) reported that one to two babies out of every 1000 live births are identified with Down syndrome (DS). The increased longevity of PWDS is of concern as this increases the demands placed on parents and caregivers who act as resident guardians (Barnhart & Connolly 2007). Because of the generally poor cardiometabolic risk profile and aerobic capacity of PWDS, they are doubly dependent on their caregivers for whom the burden of care only increases as both caregivers and patients age.

Patients with Down syndrome have an increased risk of acquiring secondary physiological pathologies due primarily to a physically inactive lifestyle and poor nutritional choices (Heller et al. 2008). These pathophysiological conditions include cardiovascular diseases, pulmonary hypoplasia, muscle hypotonia, osteoporosis, arthritis, osteoarthritis, diabetes mellitus and obesity (Heller et al. 2008). Muscle atrophy as well as poor muscle strength and endurance are co-maladies of physically inactive (sedentary) living and are frequently observed in PWDS (Dishman, Heath & Lee 2013). Heller et al. (2008) reported that the average longevity of PWDS is 55 years – 11 years fewer than individuals with other intellectual disabilities and 15 years shorter than the general populace. Rimmer, Braddock and Fujiura (1995) reported that only 10% of intellectually disabled individuals engage in a minimum of 3 days of physical activity weekly; the sedentary lifestyle adopted by the majority of intellectually disabled individuals adversely contributes to their poor fitness status, further contributing to the higher rates of obesity that are found among PWDS.

The cardiovascular diseases that have been identified among PWDS include mitral value prolapse, endocarditis, atherosclerosis and congestive heart failure (Vis et al. 2009). Patients with Down syndrome have an incidence of obesity that ranges from 31% to 47%; the high level of obesity has been associated with their sedentary lifestyle and poor eating habits (Vis et al. 2009). Abnormally high lipid profiles among PWDS are correlated with atherosclerosis (Wallen et al. 2009). The physically inactive lifestyle adopted by PWDS is furthermore associated with lower cardiorespiratory capacity, higher adiposity and reciprocal lower muscle mass, poor muscular strength, endurance, hypotonic muscles, and lower sympathetic nervous system response to physical activity and exercise (Izquierdo-Gomez et al. 2015).

Although a plethora of literature extolling the virtues of adopting a physically active lifestyle exists, few review articles describing the empirical findings of the benefits of physical activity and exercise among individuals with DS have been published (2007–2017). The commentary offered by Barnhart and Connolly (2007) suggests that as PWDS age they have higher cardiometabolic risk profiles, and that, consequently, the adoption of regular physical activity would be beneficial. The study undertaken by Barnhart and Connolly (2007) is, however, limited in so far as they failed to describe their literature gathering technique. A further limitation of the study lies in the absence of an explanation of the manner in which exercise could improve the health of PWDS.

Similarly, the clinical commentary of Fernhall, Mendonca and Bynard (2013) does not reflect the manner in which the literature was sourced; the authors, however, postulate that poor aerobic capacity in PWDS is attributed to their autonomic dysfunction. The systematic literature review by Bertapelli et al. (2016) focused on the prevalence of obesity among young PWDS, and upon interventions within this population, noting the high occurrence of obesity among young PWDS. They further highlighted the inconsistent impact of interventions that sought to curtail the obesity of young PWDS. However, Bertapelli et al. (2016) only reviewed five exercise and physical interventions, unlike this commentary, which has identified 11. This review sought to determine whether subsequent experimental studies had been conducted after the Bertapelli et al. (2016) review, and whether these studies provided evidence to substantiate the claim that regular physical activity decreases obesity among PWDS, thereby improving their cardiometabolic risk profile. The present review further sought to determine whether exercise therapy improved the cardiometabolic risk profile of PWDS, whereas Shields et al. (2018) focused on the impact of exercise induced oxidative stress on PWDS. The findings detailed by Shields et al. (2018) did not relate to the cardiometabolic risk profile of PWDS. The aforementioned empirical studies were reviewed according to Mill’s Canons to ascertain the vigour of causal relationship between regular exercise interventions and improved health among the PWDS.

This review offers two unique characteristics differentiating it from previous reviews: (1) a description of the pathogenesis of obesity, diabetes mellitus and atherosclerosis common among PWDS, and (2) a description of the physiological mechanism of how regular aerobic exercise and physical activity improve the cardiometabolic profile of PWDS.

## Methods

The literature review followed the Preferred Reporting Items for Systematic Reviews and Meta-Analyses (PRISMA) practices. This was done to ensure that all pertinent literature was sourced and synthesised into the drafting of this commentary.

### Literature surveillance

**FIGURE 1:**
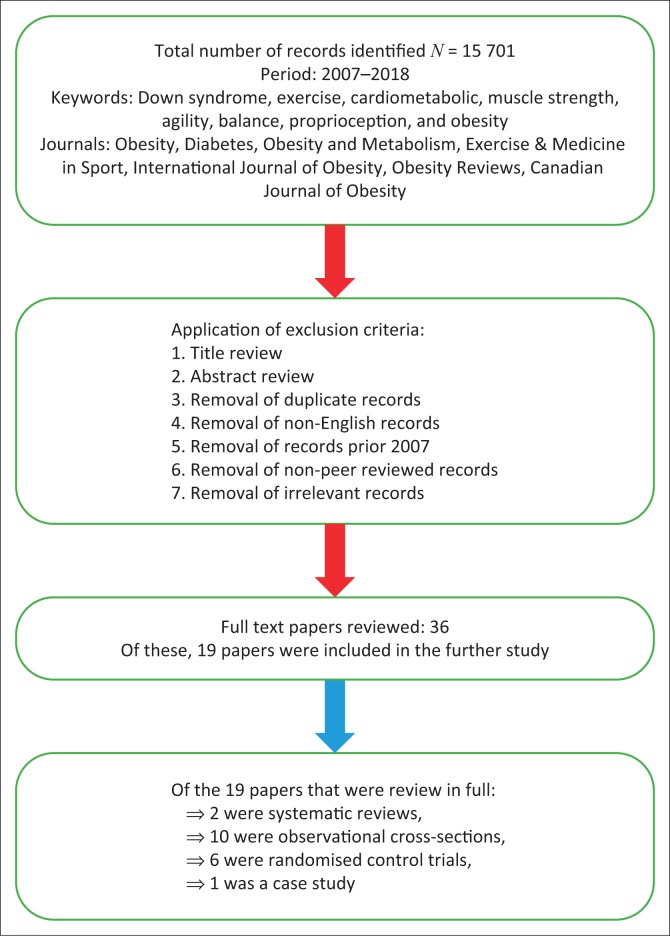
Conceptualisation of the review process.

An exploration of peer-reviewed literature within the Crossref metadatabase was completed. The Crossref metadatabase is an educational databank, which is composed of the PubMed, Science Direct, Ebscohost, CINAHL and Google Scholar search engines (Figure 1). The keywords used in the literature search were Down syndrome, exercise, cardiometabolic, muscle strength, agility, balance, proprioception and obesity. The selection criteria of the literature were accomplished in the subsequent three phases: (1) title review, (2) abstract review and (3) full text review. The records were screened by T.J.E. and Barnard M.B.

### Admissibility standards

Participants were records pertaining to PWDS and exercise. The interventions were the recorded results of exercise interventions on the cardiometabolic risk profile, aerobic capacity and muscle strength of PWDS. Applicable findings included (1) the influence of exercise on the cardiometabolic profile of PWDS, (2) the influence of exercise on the muscle strength and endurance of PWDS and (3) aerobic capacity (the effect of exercise on the ease of performing daily activity of PWDS). The elimination benchmarks included (1) the literature preceding 2007, (2) evidence relating to exercise with regard to individuals with other intellectual disabilities, (3) the literature related to the impact of medical, pharmaceutical, nutritional and psychological interventions among PWDS, (4) non-English articles and (5) the literature concerning the impact of exercise on physiological, cognitive and behavioural aspects outside the domain of the prescribed outcome interests of this article.

### Evaluation of records

The literature was reviewed according to the suitability of the title and adherence of each article to the inclusion criteria. The merit of each record was evaluated using a modified Downs and Black Appraisal Scale which inspects the value of randomised controlled trials, non-randomised, pseudo-randomised controlled articles, comparative studies with and without concurrent controls, and case series and/or studies with either post-test or pre-test/post-test outcomes (Downs & Black 1998) (Tables 1 and 2). These measures were undertaken to eradicate researcher bias. The modified checklist comprises 16 questions with a maximum score of 16 points. Answers were given a score of either 0 (no) or 1 (yes). The questions adopted from the Downs and Black Appraisal Scale were 1, 3, 4, 5, 6, 10, 11, 12, 13, 14, 18, 20, 21, 22, 23 and 27. These questions were categorised into four subdivisions, which evaluate the whole value of each record (Table 2). The categorising included reporting prowess (*n* = 6 questions), external validity (*n* = 3 questions), internal validity (*n* = 6 questions) and power of significance (*n* = 1 questions) (Downs & Black 1998). All authors were able to query the scoring of each record and would then discuss the scores adopting the jointly accepted score. The summation of these scores was then transformed to a percentage to rate the overall value of the individual records (Downs & Black 1998). The overall value of the records was categorised using a scale demarcated as follows: < 50% (weak), 50% – 69% (fair), 70% – 79% (good) and ≥ 80% (very good) (Downs & Black 1998). The mean rating of the selected papers was fair.

**TABLE 1:** Appraisal of the hierarchy of records.

Level	Type of record	No.	Authors
Level I	Systematic reviews and clinical commentaries	2	Bertapelli et al. (2016), Shields et al. (2018)
Level II-1	Randomised controlled trials	6	Shields and Taylor (2010), Gupta and Singh (2011), Ulrich et al. (2011), Lin and Wuang (2012), Ordonez et al. (2012), Silva et al. (2017)
Level III-1	Pseudo-randomised controlled trial	0	
Level III-2	Comparative study with concurrent controls	0	
Level III-3	Comparative study without concurrent controls	10	Ordonez and Rosety-Rodriguez (2007), Aguiar et al. (2008), Flore et al. (2008), Fernhall et al. (2009), Rosety-Rodriguez et al. (2010), Izquierdo-Gomez et al. (2015), Wee et al. (2015), Krause et al. (2016), Izquierdo-Gomez et al. (2017), Shields et al. (2017)
Level IV	Case series/studies with either post-test or pre-test/post-test outcomes	1	Berg et al. (2012)

**TABLE 2:** Evaluation of records.

Authors	Downs and Black appraisal					
	Reporting (*n* = 6)	External validity (*n* = 3)	Internal validity (*n* = 6)	Power (*n* = 1)	Total (*n* = 16)	Grading % = *x*/16 × 100
Ordonez and Rosety-Rodriguez (2007)	6	2	2	1	11	68.7
Aguiar et al. (2008)	5	2	2	1	10	62.5
Flore et al. (2008)	5	2	4	1	12	75.0
Fernhall et al. (2009)	5	2	4	1	12	75.0
Rosety-Rodriguez et al. (2010)	6	2	2	1	11	68.7
Shields and Taylor (2010)	5	2	5	1	13	81.2
Gupta and Singh (2011)	5	3	4	1	13	81.2
Ulrich et al. (2011)	5	3	5	1	14	87.5
Berg et al. (2012)	5	3	2	1	11	68.7
Lin and Wuang (2012)	5	2	5	1	13	81.2
Ordonez et al. (2012)	5	2	6	1	14	87.5
Izquierdo-Gomez et al. (2015)	5	2	4	1	12	75.0
Wee et al. (2015)	5	1	4	1	11	68.7
Bertapelli et al. (2016)	4	0	2	1	7	43.7
Krause et al. (2016)	4	2	4	1	11	68.7
Izquierdo-Gomez et al. (2017)	4	2	4	1	11	68.7
Silva et al. (2017)	5	1	2	1	9	65.2
Shields et al. (2018)	5	0	2	1	8	50.0

### Ethical consideration

This article is an overview or clinical commentary; therefore, no subjects were involved.

## Results

A total of 1331 participants were enrolled across the 19 studies with a mean age of 18.1 ± 6.8 years, a mean body mass of 61.8 kg ± 13.3 kg, a mean height of 1.53 ± 0.09 m and a mean BMI of 25.2 kg/m^2^ ± 4.0 kg/m^2^. The 19 studies comprised 2 systematic reviews, 1 case study, 10 observational cross-sectional and 6 randomised controlled trials (Table 1). Eleven studies reviewed the influence of exercise and physical activity on the health status of PWDS. Regular aerobic exercises reduced lipid peroxidation, oxidative stress, arterial cell wall damage and body fat all while enhancing insulin sensitivity, which was favourably associated with lowering the metabolic risk profile of participants. Regular muscle strengthening improved lower limb strength and improved daily habitual activities (walking upstairs and grocery shelving), motor skills and posture (Table 3).

**TABLE 3:** Sequential summary of the characteristics and conclusions of the records (*n* = 19).

Authors (country)	Characteristics of the study			
	Type of study	Sample	Method	Findings
Ordonez and Rosety-Rodriguez (2007) Spain	Observational cross-sectional	31 male adolescent PWDS, age = 16.3 years, body mass = 70.8 kg, height = 1.55 m, BMI = 29.3 kg/m^2^	All participants completed a 12 week, 60 min aerobic training programme at 60% – 75% peak heart rate on a treadmill. Blood samples were drawn to identify malondialdehyde (MDA) levels	Aerobic exercise significantly lowers lipid peroxidation as reflected through MDA levels
Aguiar et al. (2008) Brazil	Observational cross-sectional	*N* = 21 PWDS, age = 23.3 years, BMI = 23.0 kg/m^2^	The physical activity intervention was comprised of supervised judo training of a controlled intensity for 16 weeks (three 50-min sessions per week). Blood lactate, lipid peroxides and carbonyls were measured to determine oxidative stress	PWDS experience the oxidative stress benefits of physical activity, which may positively influence their unhealthy cardiometabolic risk profile
Flore et al. (2008) Belgium	Observational cross-sectional	Group 1 (PWDS): *N* = 13 (age = 22 years, body mass = 63.1 kg, height = 1.6 m, BMI = 24.6 kg/m^2^, fat mass = 19.9%, VO_2max_ = 44.4 kg/mL/min) Group 2 (non-DS): *N* = 15 (age = 22 years, body mass = 67.6 kg, height = 1.7 m, BMI = 23.3 kg/m^2^, fat mass = 13.5%, VO_2max_ = 60.8 mL/kg/min)	All participants’ anthropometric measures and blood profiles were recorded. Each participant completed an individualised maximal oxygen (VO_2max_) consumption test on a treadmill. The treadmill test determined VO_2max_ and peak heart rate (HR_peak_). Once a comfortable walking or running pace was determined, the slope was increased by 2% every minute until the participants reported exhaustion. Gas exchange and heart rate were continuously recorded. Body mass and height were measured to calculate participants’ BMI. Total body fat was derived measuring skinfold measurement at the biceps, triceps, supra-iliac and sub-scapular anatomical sites. Waist and hip circumferences were measured to determine visceral fat	Although the PWDS displayed greater oxidative stress and lower insulin sensitivity, this was not conclusively associated with metabolic syndrome
Fernhall et al. (2009) United States	Observational cross-sectional	Group 1 (PWDS): *N* = 20 (age = 24 years, body mass = 70.6 kg, height = 1.56 m, BMI = 28.8 kg/m^2^, VO_2peak_ = 27.3 mL/kg/min, HR_peak_ = 170 bpm) Group 2 (PWDS): *N* = 21 (age = 26 years, body mass = 69.9 kg, height = 1.70 m, BMI = 24.3 kg/m^2^, VO_2peak_ = 40.7 mL/kg/min, HR_peak_ = 189 bpm)	All participants’ guardians completed a healthy screening questionnaire. Maximal oxygen (VO_2peak_) consumption and peak heart rate (HR_peak_) were determined through a peak cardiopulmonary test conducted using a treadmill based individualised protocol	The poor catecholamine (epinephrine and norepinephrine) response to peak exercise among PWDS suggests that this may be the principle reason for their low peak heart rates and poor aerobic capacity during exercise
Rosety-Rodriguez et al. (2010) Spain	Observational cross-sectional	31 male adolescent PWDS, age = 16.3 years, body mass = 70.8 kg, height = 1.55 m, BMI = 29.4 kg/m^2^	All participants completed a 12 week 60 min aerobic training programme at 60% – 75% peak heart rate on a treadmill. The training session was composed of a 15 min warm-up component followed by 20–35 min exercise (initially beginning at 20 min, thereafter increasing 5 min each third week) at a work intensity of 60% of peak heart rate (which similarly increased by 5% each third week) and which was followed by a 10 min cool-down period. Blood samples were drawn to identify plasma allantoin levels	The findings indicate that regular aerobic training lowers plasma allantoin levels and improves antioxidant enzyme activity, reducing lipoperoxidation
Shields and Taylor (2010) Australia	Randomised controlled trial	Group 1/experimental: *N* = 11, age = 15.9 years, body mass = 63 kg, height = 1.59 m, BMI = 25.5 kg/m^2^, number of boys (*n* = 8) and girls (*n* = 3) Group 2/control: *N* = 12, age = 15.3 years, body mass = 58 kg, height = 1.56 m, BMI = 24.0 kg/m^2^, number of boys (*n* = 9) and girls (*n* = 3)	The experimental group undertook a 10-week resistance strength programme, which included six exercises performed twice a week for 6 weeks. The experimental group completed six exercises using weight machines: three for the upper limbs (latissimus dorsi pull-down, seated chest press, seated row) and three for the lower limbs (seated leg press, knee extension, calf raise). The control continued with their normal daily activities. Pre- and post-tests included muscle strength 1 repetition maxim (1 RM), a timed stair climb test and a grocery shelving task	The experimental group increased their lower limb strength (*p* < 0.05), but no difference was seen in upper limb strength, timed stair climb, and grocery shelving task. These findings indicate that progressive resistance training can be safely used to increase the lower limb strength of PWDS
Gupta and Singh (2011) India	Randomised controlled trial	Group 1/experimental: *N* = 12, age = 13.5 years, IQR = 36–52, body mass = 28.4 kg, height = 1.32 m, BMI = 16.2 kg/m^2^ Group 2/control: *N* = 11, age = 13 years, IQR = 38–49, body mass = 23.9 kg, height = 1.37 m, BMI = 12.7 kg/m^2^	The experimental group followed a lower limb resistance-strengthening programme with balance exercises. The control group continued with their normal daily activities. Body mass and height were measured. A dynamometer was used to assess the strength of hip flexors/extensors, abductors/adductors, knee flexors/extensors and ankle plantar flexors. The experimental group completed a specific 6-week exercise training programme that was composed of progressive resistance lower limb exercises and proprioception training. Strength training began at 50% of participant’s 1 RM. Resistance exercises were targeted to strengthen hip flexors, abductors, extensors, knee flexors, extensors and ankle plantar flexors. Proprioceptive exercises included horizontal and vertical jumps, stalk stand with eyes open, tandem stance, walking online, walking on balance beam and jumping on a trampoline	The experimental group demonstrated significant lower limb strength gains and improved balance (*p* < 005) as compared to the control group. The findings suggest that specific exercises can improve the strength and balance of PWDS
Ulrich et al. (2011) United States	Randomised controlled trial	Group 1/experimental: *N* = 36, age = 12.0 years, body mass index = 24.3 kg/m^2^, percentage body fat = 36.7%, time performing moderate to vigorous physical activity = 39.2 min, diagnosed with DS Group 2/control: *N* = 36, age = 12.4 years, BMI = 23.0 kg/m^2^, percentage body fat = 32.1%, time spent performing moderate to vigorous physical activity = 46.9 min, diagnosed with DS	All participants wore an accelerometer to measure physical activity and DXA measured body fat. 56% of the experimental group learned to ride a bicycle within the 5-day intervention. The motor skill acquisition of learning to ride the bicycle encouraged subjects to become more involved in moderate to vigorous physical activity, thereby decreasing their body fat percentage 12 months post-intervention (*d* = 0.47)	Children diagnosed with DS can learn to ride a two-wheel bicycle, demonstrating proficient motor skills and proprioception. The ability to ride a bicycle encouraged children to engage in moderate and vigorous physical activity thereby favourably reducing their body fat percentage
Berg et al. (2012) United States	Case study Observational	A 12-year-old child diagnosed with DS and no previous experience with a Nintendo Wii	The child self-selected the Nintendo Wii games, which were played 4 times a week for 8 weeks. Each session lasted 20 min. Pre-test included postural stability, limits of stability and the Bruininks-Oseretsky Test of Motor Proficiency	The child’s persistent use of Nintendo Wii games produced improved motor skills and postural stability. These findings suggest that the Nintendo Wii games could be an effective physical activity tool in order to encourage persistent physical activity and improve motor skills and postural stability
Lin and Wuang (2012) Taiwan	Randomised controlled trial	Group 1/experimental: *N* = 46, age = 15.6 years, IQR = 52, body mass = 57.2 kg, height = 1.53 m, number of girls = 25 and boys = 21 Group 2/control: *N* = 46, age 14.9 years, IQR = 53, body mass = 58.8 kg, height = 1.51 m, number of girls (*n* = 24) and boys (*n* = 22).	The experimental group completed an exercise training programme that comprised 5 min of treadmill walking and a 20-min virtual reality-based activity, three sessions/week for 6 weeks. Pre- and post-testing included agility (Bruininks-Oseretsky Test of Motor Proficiency) as well as muscle strength (hip flexion/extension and hip abduction, knee flexion/extension and plantar flexion)	The experimental group had significantly improved agility and muscle strength (*p* < 0.05 and *d* = 0.5–0.8). These findings indicate that short-term exercise training can improve the muscle strength and agility of adolescents with DS
Ordonez et al. (2012) Spain	Randomised controlled trial	Group 1/experimental: *N* = 31, male, age = 16.3 years, height = 1.66 m, body mass = 78.7 kg, body mass index = 28.5 kg/m^2^, per cent body fat = 31.8% Group 2/control: *N* = 7, age, gender and BMI matched. Plasma carbonyl content was determined pre- and post-intervention	The experimental group completed a 12-week aerobic programme on a treadmill (warm-up: 15 min, followed by: 20–35-min exercise at 60% – 75% maximum heart rate with a 10 min warm-down)	The principal findings of this study indicate that a 12-week aerobic programme significantly reduced protein oxidation among PWDS (*p* < 0.05)
Izquierdo-Gomez et al. (2015) Spain	Observational cross-sectional (up and down study)	Group 1 (PWDS): *N* = 100 (age = 15.4 years, body mass = 52.6 kg, height = 1.48 m, BMI = 23.7 kg/m^2^, waist circumference = 73.6 cm, percent body fat = 34.8%). Group 2 (non-DS): *N* = 100 (age = 13.5 years, body mass = 54.9 kg, height = 1.61 m, BMI = 20.8 kg/m^2^, waist circumference = 68.2 cm, body fat percentage = 19.6%)	All participants completed the ALPHA health-related fitness test to measure fitness and fat levels. Muscular fitness was measured using the handgrip strength and the standing long jump test. Adolescents were instructed to wear the accelerometer for seven consecutive days. Adolescents had to have at least 3 days of valid data with a minimum of 8 h of data. Body mass and height were measured, from which the participant’s BMI was calculated. Body fat percentages were calculated from triceps and sub-scapular skinfold thicknesses using the Slaughter equations	Adolescent PWDS had higher fat levels and lower fitness status as compared to adolescents without DS (*p* < 0.05). Fat levels were not linked to physical activity status; however, higher physical activity levels were positively associated with aerobic fitness status
Wee et al. (2015) United States	Observational cross-sectional	Group 1 (PWDS): *N* = 151 (males: 57 and females: 47), age = 21 years, body mass = 64.8 kg, height = 1.53 m, BMI = 27.4 kg/m^2^ Group 2 (non-DS participants): *N* = 180 (males = 131, females = 49), age = 21 years, body mass = 64.6 kg, height = 1.63 m, BMI = 23.9 kg/m^2^	All participants underwent a treadmill test to determine VO_2peak_ (peak maximal oxygen consumption) and HR_peak_ (peak heart rate during exercise), body mass and height measurements were also taken (BMI)	PWDS have low VO_2peak_ and HR_peak_ irrespective of the presence of obesity and age
Bertapelli et al. (2016) Brazil and United States	Systematic review	Participants were empirical records of the prevalence, determinants and consequences of as well as interventions in overweightness and obesity among children and adolescents with DS	A literature search was conducted using the following search engines: MEDLINE, Embase, Web of Science, Scopus, CINAHL, PsycINFO, SPORTDiscus, LILACS and COCHRANE	Youth with DS have a high prevalence of overweight and obesity compared to youth without DS. Increased leptin levels, poor nutritional plans, decreased resting energy expenditure, comorbidities and low physical activity levels are determinants of obesity among PWDS. Obesity was confidently linked to dyslipidaemia, obstructive sleep apnea and gait disorder. Interventions for obesity prevention and control included exercise-based programmes, which did not achieve sufficient results
Krause et al. (2016) Australia	Observational cross-sectional	Total number of participants (*N* = 261, DS: *N* = 119, non-DS (but with other intellectual disabilities): *N* = 42), males (145) and females (*N* = 116) of total sample, DS participants overweight (*N* = 49), DS participants obese (*N* = 45). BMI was the criterion used to identify overweight and obesity	A cross-sectional survey in addition to a medical record review of age, gender, body mass, height, pathology, mobility and medication concerning 261 adolescents with DS was conducted. Body mass index was used to categorise participants’ as normal/underweight, overweight or obese according to the International Obesity Taskforce definitions. 22.4% and 20.6% of DS participants were overweight and obese respectively; this is in comparison to 33.3% and 35.7% of the non-DS participants	These findings indicate that overweight and obesity is a common problem challenging both PWDS and individuals with other intellectual disabilities
Izquierdo-Gomez et al. (2017) Spain	Observational cross-sectional longitudinal	Total number of participants (girls *n* = 38, boys *n* = 61). The objective of the study was to measure physical activity profiles 2 years after the initial up and down study. Mean age 15.9 ± 2.4 years	All participants completed the ALPHA health-related fitness test to measure fitness and fat levels. Muscular fitness was measured using the handgrip strength and the standing long jump test. Adolescents were instructed to wear the accelerometer for seven consecutive days. Adolescents had to have at least 3 days of valid data with a minimum of 8 h of data. Body mass and height were measured, from which the participants’ BMI was calculated. Body fat percentages were calculated from triceps and sub-scapular skinfold thicknesses using the Slaughter equations	From the initial study only 17% of the cohort maintained the specified physical activity guidelines. This indicates that adolescents PWDS need to be continuously encouraged to be physically active
Shields et al. (2017) Australia	Observational cross-sectional	14 PWDS aged 12.9 years (6 girls and 8 boys), height = 1.41 m, body mass = 51.8 kg, BMI = 25.0 kg/m^2^, waist circumference = 79.5 cm, VO_2max_ = 39.6 mL/kg/min, peak heart rate = 180 bpm, level of IQ perceived: mild = 3, mild to moderate = 8, moderate = 3	Cardiovascular fitness was assessed through the Fernhall and Tymeson protocol, where the participant walked or ran on a treadmill wearing a heart rate monitor. The treadmill pace incrementally increased, while simultaneously measuring oxygen uptake through a portable Oxycon mobile system. Physical activity volume was measured with an accelerometer over an 8-day period. A day was considered valid when a child wore the monitor for at least 10 h. Anthropometric measures to determine BMI and waist circumference were taken and complied with the Cameron protocol	PWDS who were aerobically fitter had smaller waist circumferences (*r* = 0.7) and lower BMI (*r* = 0.7). Evidence indicates that body composition is inversely associated with aerobic fitness
Silva et al. (2017) Portugal	Randomised controlled trial	Group 1/experimental: *N* = 12 PWDS Group 2/control: *N* = 13 PWDS Ages ranged from 18 to 60 years, mean body mass = 71 kg, height = 1.51 m, BMI = 32.1 kg/m^2^	The experimental group completed a 2-month Wii-based physical activity intervention of three 60-min sessions per week. The intervention included aerobic, balance and strengthening components. All participants underwent an anthropometric assessment, physical fitness, motor proficiency and Bruininks-Oseretsky functional mobility tests. The control group continued with their normal daily activities. Body mass, BMI, body fat percentage, visceral fat levels and muscle mass were obtained using a segmental body composition analyser (Tanita BC 531). Physical fitness was assessed by use of the Eurofit Test Battery that measured speed of limb movement, handgrip strength, running speed and agility, balance, flexibility, standing broad jump, trunk strength, muscular endurance and a 6-min walk	The Wii-based physical activity intervention proved to be a successful instrument for the improvement of the physical fitness of PWDS, further improving their aerobic capacity, motor proficiency, functional mobility and lower strength as well as their lower body composition (*p* < 0.05). These findings show that exergaming may offer a valuable physical intervention strategy
Shields et al. (2018) Australia	Systematic review	Participants were empirical studies pertaining to exercise induced oxidative stress among PWDS	The authors adopted the PRISMA guidelines in order to gather eligible papers. Six electronic databases (Medline, EMBASE, CINAHL, PubMed, AMED and SPORTDiscus) were searched	Uncertainty remains regarding the effect of exercise on oxidative stress in PWDS

PWDS, patients with Down syndrome; BMI, body mass index; MDA, malondialdehyde; HR, heart rate; RM, repetition maxim; IQR, intelligence quotient range; DXA, dual energy X-ray.

## Discussion

The discussion of findings will follow the interest outcomes of the literature search, namely the impact of exercise on the cardiometabolic profile of PWDS, as well as on their muscle strength, agility and balance. The discussion of the effects of exercise on the cardiometabolic profile of PWDS will detail the role of oxidative stress on the pathogenesis of diabetes mellitus and atherosclerosis and underline the favourable impact of exercise on the improvement of the cardiometabolic profile of PWDS. Finally, the articles synthesised into this commentary will be reviewed according to Mill’s Canons (Dishman et al. 2013) so as to ascertain the strength of the relationship between exercise and the change in health status of PWDS.

### Impact of exercise on the cardiometabolic profile of patients with Down syndrome

Patients with Down syndrome have a high incidence of metabolic syndrome, which has been related to heightened cardiometabolic risk profiles (diabetes mellitus, poor insulin sensitivity and high insulin resistance, obesity, atherosclerosis, high low-density lipoprotein cholesterol, hypertension and poor aerobic capacity) (Wallen et al. 2009). Furthermore, high oxidative stress has been related to elevated insulin resistance, poor insulin sensitivity, atherosclerosis and hypertension (Flore et al. 2008). Oxidative stress impairs β-cell function, which reduces the production of insulin by impairing glucose-stimulated insulin secretion, thereby creating a state of hyperglycaemia, which ultimately leads to the development of diabetes mellitus (Tangvarasittichai 2015). Abnormal lipid metabolism has been related to premature risk for the development of atherosclerosis among PWDS (Vis et al. 2009). Aguiar et al. (2008) and Silva et al. (2017) reported that consistent physical activity or exercise lowers body fat, suggesting that regular exercise and physical activity can improve the cardiometabolic risk profile of PWDS. One of the principal benefits of regular aerobic exercises is the use of fats (lipids) for energy, reducing fat content and thereby improving the individual’s cardiometabolic risk profile (Durstine et al. 2011). A further benefit of regular aerobic exercise is the earlier use of lipids, thereby conserving muscle and liver glycogen stores, which has a carbohydrate sparing effect. The use of lipids as an energy fuel yields more ATP than the decomposition of carbohydrates. The catabolism of lipids occurs through the process of lipolysis. The more lipids used, the greater the reduction in fat stores, thereby lowering body fat (McArdle, Katch & Katch 2000). Ulrich et al. (2011) reported that PWDS who exercise regularly reduce body fat, but the authors did not explain the exercise induced physiological mechanism behind the fat loss and how this relates to obesity. Aguiar et al. (2008) reported on the oxidative stress benefits of physical activity, which may positively influence unhealthy cardiometabolic risk profiles, but did specifically explain its use in combating obesity. The study undertaken by Aguiar et al. (2008) was not a randomised control, thereby limiting the significance of the findings and Ulrich et al. (2011) in a single randomised controlled study further showed the paucity of validation in the aforementioned studies. More empirical randomised controlled studies that discuss the biochemical exercise induced mechanism of fat loss relating to reduction in obesity levels among PWDS are therefore required to validate these findings. Such studies would further encourage PWDS and their caregivers to become involved in regular physical activity and exercise.

### Impact of aerobic exercise on the pathogenesis of atherosclerosis

Patients with Down syndrome have been identified as having high oxidative stress, which serves as a pathogenic mechanism for the development of atherosclerosis, neurodegeneration, cell aging, cancer and immunological disorders (Ordonez & Rosety-Rodriguez 2007). Oxidative injuries in cardiovascular tissue such as arteries provide opportunities for the development of atheroma (cholesterol plaque), facilitating the pathogenesis of atherosclerosis (the build-up of low density lipoprotein cholesterol, fat, triglycerides and macrophages forming an atheroma/plaque, which reduces blood flow) and arteriosclerosis (the process whereby the arterial walls thicken and harden, losing their elasticity and reducing blood flow) (Tangvarasittichai 2015). Regular endurance exercise and physical activity decrease lipid peroxidation and arterial cell wall damage, which limits the pathogenesis of atheroma (Rosety-Rodriguez et al. 2010). Regular exercise furthermore facilitates the release of nitrate oxide, vasodilating blood vessels and thereby enhancing blood flow (Durstine et al. 2011). These findings demonstrate the benefit of regular aerobic exercise for the reduction of the cardiometabolic risk profile of PWDS. However, more experimental investigations are required to validate the findings of Rodriguez et al. (2010), among PWDS.

### Aerobic capacity of patients with Down syndrome

Fernhall et al. (2013) and Wee et al. (2015) indicated that PWDS have low aerobic capacity, characterised by low VO_2max_ and peak heart rates. Fernhall et al. (2013) postulate that autonomic dysfunction is the principal contributor to the poor aerobic capacity and maximal oxygen consumption of PWDS, which may lead to their poor cardiometabolic risk profiles. The poor catecholamine (epinephrine and norepinephrine) response to peak exercise among PWDS suggests that this may be the principal reason for the low peak heart rates and poor aerobic capacity during exercise of PWDS (Fernhall et al. 2009). Shields et al. (2017) reported that PWDS who were aerobically fitter had smaller waist circumferences and a lower BMI. Although the evidence provided by Shields et al. (2017) suggests that body composition is inversely associated with aerobic fitness, the research study design (observational) limits the significance of the findings. Despite this, the findings of Shields et al. (2017) nevertheless motivate exercise interventions for PWDS. Silva et al.’s (2017) study is the only randomised controlled study that was included in this review; the study demonstrates that regular aerobic exercise increased the aerobic capacity of PWDS and thus warrants validation through more randomised controlled trials. Although the physiological mechanism has been identified, more empirical investigations are required to determine the manner in which the aerobic capacity of PWDS can be improved. This data can then, in turn, assist in the improvement of the cardiometabolic risk profile of PWDS.

### The effect of exercise on the muscle strength, agility and balance of patients with Down syndrome

Patients with Down syndrome have poor muscle strength, agility and balance as compared to similarly age-matched peers (Izquierdo-Gomez et al. 2015). Shields and Taylor (2010) and Gupta and Singh (2011) reported that regular strengthening exercises improve the muscle strength of PWDS. Shields and Taylor (2010) also demonstrated that the increase in muscle strength served to enhance both daily functional activities (such as walking upstairs with greater ease) and the performance of rudimentary tasks (such as packing shelves). Gupta and Singh (2011) illustrated that regular strength training improves muscle strength and proprioception (balance). However, Gupta and Singh (2011) did not explain how improved muscle strength was associated with improved balance. Empirical investigations explaining the relationship between the improvement in muscle strength and the consequent improvement in balance in PWDS are advised. Silva et al. (2017) also reported that computer games (namely, the Nintendo Wii) assisted in increasing lower limb muscle strength, aerobic capacity and motor coordination. The merit of the above-mentioned studies is their randomised controlled design that validates each other’s findings: regular resistance training- and physical activity-based electronic games do have the potential to improve muscle strength and balance, thereby providing daily functional benefits. The effects of different types of strength training and exercises, such as circuit training, swimming and basic plyometric drills on the health profile of PWDS, should be investigated through randomised controlled trials. The adoption of alternate strengthening activities, such as circuit training, plyometric and swimming, will add variety to the exercise regime of PWDS, thereby helping to maintain adherence to exercise therapy. Furthermore, regular resistance training does have cardiometabolic benefits, which needs to be explored in the context of this particular population, in so far as obesity is associated with the development of diabetes mellitus. Regular strength training has been reported to lower insulin resistance and improve insulin sensitivity, thereby improving the cardiometabolic profile of diabetic patients (Durstine et al. 2011). Although these findings are yet to be validated for PWDS, they show promise in so far as strengthening can also serve to improve the cardiometabolic risk profiles of PWDS. More empirical randomised controlled trails are, however, needed. Over and above this, Gupta and Singh (2011) and Berg et al. (2012) have documented that regular exercise can improve postural stability among PWDS.

### Strength of evidence supporting the beneficial effects of regular exercise in improving the cardiometabolic risk profile, muscle strength and proprioception of patients with Down syndrome

The authors adopted Mill’s Canons (Dishman et al. 2013) to determine the strength of evidence supporting the causal inference relating to the impact of exercise interventions on chronic diseases. Mill’s Canons have the following five criteria:

- Temporal sequence refers to the sequence of the exposure of the intervention, which must precede the change of the diseased condition within a sufficient time frame to make a plausible conclusion. Ten of the 11 studies demonstrated that regular exercise improves the health status of PWDS. Six of these 11 studies focused on the effect of exercise on the cardiometabolic risk profile of PWDS. Five studies (83.3%) out of the six indicated that regular exercise improves the cardiometabolic risk profile of PWDS (Aguiar et al. 2008; Ordonez & Rosety-Rodriguez 2007; Ordonez et al. 2012; Rosety-Rodriguez et al. 2010; Silva et al. 2017). Of the five studies, two were randomised control studies (Ordonez et al. 2012; Silva et al. 2017) and three were observational cross-sectional studies (Aguiar et al. 2008; Ordonez & Rosety-Rodriguez 2007; Rosety-Rodriguez et al. 2010). Four randomised control trials indicated that regular exercise improves the strength and proprioception of PWDS, thereby addressing the patients’ hypotonic muscle deficits (Gupta & Singh 2011; Lin & Wuang 2010; Shields & Taylor 2010; Silva et al. 2017). Shields and Taylor (2010) also associated increased muscle strength with improved daily functional activities such walking and packing shelves among PWDS. Two randomised controlled trials and one case study demonstrated that regular physical activity improves the postural stability of PWDS (Berg et al. 2012; Gupta & Singh 2011; Ulrich et al. 2011).
- Strength of association refers to the clinical meaningful difference between the disease and the intervention. Ten of the 11 studies indicated a strong association between the exercise and physical activity interventions and improved cardiometabolic risk profile, strength, proprioception and postural balance (Table 3).
- Consistency of results refers to the consistent observation of the association between the outcome of the intervention and the disease. Of the 11 studies reviewing the effect of regular exercise and physical activity on the health status of PWDS, 10 indicated positive outcomes (90.9%) (Table 3).
- Biological plausibility refers to the clinical explanation of the observed outcome of the intervention in regard to disease. The 10 studies that showed positive outcomes described plausible explanations for these improvements (Table 3).
- Dose–response refers to the volume of intervention required to produce a specific outcome on the disease. There is, however, no consensus pertaining to the amount or volume of exercise and physical activity needed to produce beneficial outcomes. It is advised that further research investigating the dose–response concerning intensity, duration and frequency of exercise interventions and physical activity on PWDS. This new research will help medical practitioners and exercise therapists determine the adequate dose response to exercise.

## Conclusion

Clinical evidence has indicated that regular exercise benefits the health status of PWDS with regard to improving their body composition, aerobic capacity, muscle strength, proprioception and postural stability. The benefits of augmented aerobic work capacity and body composition help to lower the cardiometabolic risk profile of PWDS. However, more randomised controlled trials are needed to both determine the dose–response to exercise and validate these preliminary empirical findings. Additional empirical randomised controlled studies, which discuss the biochemical exercise induced mechanism of fat loss relating to a reduction in obesity levels among PWDS, are required to validate these findings. The effects of different types of exercises, such as circuit training, swimming and basic plyometric drills on the health profile of PWDS, should be investigated through randomised controlled trials. This review showed that aerobic exercises were primarily selected to alter the cardiometabolic profile of PWDS. Resistance training has also improved the diabetic profile of patients, and this should be explored among PWDS as well.
